# Yield Performance and Phytochemical Stability of ‘Comet’ Hop Under Contrasting Light Supplementation Regimes in Subtropical Conditions

**DOI:** 10.3390/plants14223516

**Published:** 2025-11-18

**Authors:** Caio Scardini Neves, Filipe Pereira Giardini Bonfim, Olivia Pak Campos, Viviany Viriato, Gustavo do Carmo Fernandes, Mariana Nunes Ferreira Cabral, Gabriel Cássia Fortuna, Sthefani Gonçalves de Oliveira, Adam N. Rabinowitz, Valéria Cristina Rodrigues Sarnighausen, Elizabeth Orika Ono, Júlio César Rodrigues Lopes Silva, Marcia Ortiz Mayo Marques

**Affiliations:** 1 Departamento de Produção Vegetal, Setor de Horticultura, Faculdade de Ciências Agronômicas, Universidade Estadual Paulista (Unesp), Botucatu 18610-034, SP, Brazil; olivia.pakc@gmail.com (O.P.C.); viviany.viriato@unesp.br (V.V.); gc.fernandes@unesp.br (G.d.C.F.); mariana.nf.cabral@unesp.br (M.N.F.C.); gabriel.cassia.fortuna@gmail.com (G.C.F.); elizabeth.o.ono@unesp.br (E.O.O.); 2 Centro de Pesquisa e Desenvolvimento de Recursos Genéticos Vegetais, Instituto Agronômico (IAC), Campinas 13020-902, SP, Brazil; juliocesarls2010@gmail.com (J.C.R.L.S.); marcia.marques@sp.gov.br (M.O.M.M.); 3 Agricultural Economics Department, Auburn University, Auburn, AL 36849, USA; sthefanigoncalves@hotmail.com (S.G.d.O.); adam.rabinowitz@auburn.edu (A.N.R.); 4 Departamento de Bioprocessos e Biotecnologia, Faculdade de Ciências Agronômicas, Universidade Estadual Paulista (Unesp), Botucatu 18610-034, SP, Brazil; valeria.sarnighausen@unesp.br

**Keywords:** *Humulus lupulus* L., photoperiod manipulation, phenology, essential oil, α- and β-acid

## Abstract

This study evaluated the agronomic performance and phytochemical stability of the ‘Comet’ hop (*Humulus lupulus* L.) under contrasting photoperiod management regimes (with and without supplemental lighting) in subtropical conditions over two consecutive crop cycles (2024–2025). The experiment, conducted at the School of Agricultural Sciences (FCA/UNESP, Botucatu, Brazil), followed a split-plot factorial design with ten replications. Supplemental lighting (50 W, 6500 K LED floodlights) extended the photoperiod to 17 h during the vegetative stage. Morphometric, phenological, and phytochemical parameters were analyzed, including α- and β-acid contents and essential oil composition by GC–MS and GC-FID. Supplemental lighting significantly increased plant height (590.9 cm), number of lateral branches (70.1), cone length (3.49 cm), and dry cone mass (374.6 g plant^−1^) while reducing the insertion height of the first cone (99.0 cm). α-Acid content increased from 9.35% to 11.92%, and essential oil content from 1.34% to 1.90%, while β-acid levels showed no significant variation. Chemical analysis identified 31 compounds, predominantly β-myrcene (65–74%) and sesquiterpenes such as (*E*)-caryophyllene, α-selinene, and β-selinene, exhibiting remarkable compositional stability across years and treatments. Photoperiod extension delayed floral induction, promoted biomass accumulation, and increased cone yield without altering the characteristic herbaceous–resinous aromatic profile. These findings validate supplemental lighting as a decisive strategy to optimize hop production in subtropical regions, ensuring phenological regularity, higher yield, and consistent chemical quality, thereby strengthening the viability of domestic hop cultivation in Brazil.

## 1. Introdution

**Figure 1:**
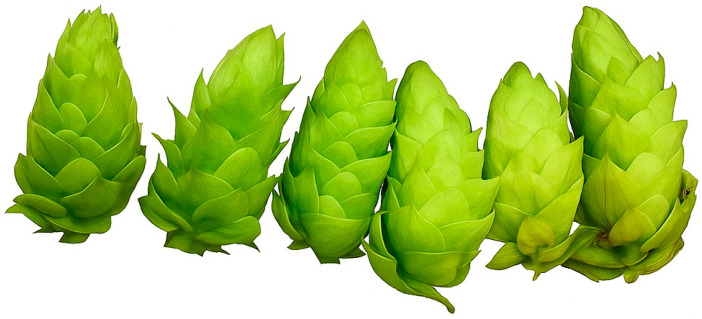
Morphological aspects of *Humulus lupulus* L. Comet variety cones, highlighting their elongated conical structure with tightly overlapping bracts and a light green to golden-green, cultivated in Botucatu, São Paulo, Brazil.

Hop (*Humulus lupulus* L.) is a perennial, dioecious horticultural plant whose economic importance lies in the female inflorescence, known as the cone (Figure 1). These cones contain lupulin glands, which are rich in resins, phenolic compounds, and essential oils, specialized metabolites of both pharmacological and industrial interest [1]. These compounds confer to hops a range of bioactive properties, including antioxidant, antimicrobial, anti-inflammatory, antimutagenic, antiallergenic, neuroprotective, and estrogenic actions [2,3]. Although hops have potential applications in the pharmaceutical industry, their primary use is in brewing, where their compounds contribute aroma, bitterness, and stability to beer [4,5].

Currently, about 97% of global hop production is destined for the brewing industry, where, in addition to providing sensory attributes, hops also exhibit antimicrobial and antioxidant activities, reducing contamination risks and improving product stability [6]. In Brazil, the brewing sector represents approximately 2.5% of the national GDP, generating around 3.1 million direct and indirect jobs and moving nearly BRL 230 billion annually [7]. Despite this major economic and social impact, Brazil remains fully dependent on imported hops, highlighting the urgent need to establish domestic production to reduce costs, strengthen the supply chain, and enhance competitiveness.

Native to the Northern Hemisphere, hops are photoperiod-sensitive plants whose flowering is triggered by decreasing day length. Flower induction occurs when the photoperiod falls below 16 h of light [8,9], explaining their adaptation to latitudes between 35° and 55° [10]. Under these conditions, plants undergo an extended vegetative phase, which is crucial for biomass accumulation and subsequent yield potential [11].

In Brazil, the latitude of Botucatu (22°50′ S) limits day length to 10–13 h between solstices, causing premature floral induction and restricting full vegetative development [12,13]. Supplemental lighting has therefore emerged as a promising strategy to manipulate the photoperiod, enabling prolonged vegetative growth and better-timed floral induction, ultimately enhancing both yield and raw material quality [11].

Released in 1974 by the United States Department of Agriculture (USDA), the Comet hop variety was developed with an emphasis on high α-acid content. Under Brazilian growing conditions, it stands out not only for its elevated levels of these compounds and well-balanced aromatic profile but also for its excellent adaptation to subtropical environments. Currently, Comet is the most widely cultivated hop in the country, combining agronomic resilience with chemical quality, attributes that enhance its potential for both bittering and aroma applications [14].

Empirical observations by Brazilian growers suggest that supplemental lighting plays a decisive role in the agronomic and phytochemical performance of hops, promoting greater vegetative vigor, increased cone production, and improved technological quality. However, systematic scientific validation is still lacking to confirm its effectiveness under subtropical conditions. In this context, the present study aimed to evaluate the productive performance and phytochemical quality of Comet plants grown under two contrasting conditions: with and without supplemental lighting.

## 2. Materials and Methods

### 2.1. Experimental Site and Climate

The experiment was conducted in the experimental and teaching orchard of the Department of Crop Science–Horticulture, Fazenda Lageado, School of Agricultural Sciences (FCA), São Paulo State University “Júlio de Mesquita Filho” (UNESP), Botucatu campus, Botucatu, SP, Brazil (22°50′ S, 48°26′ W, 791 m a.s.l.). The region is classified as Aw according to Köppen, a tropical savanna climate with a dry winter, with an annual precipitation of 1500 mm and a mean annual temperature of 21.34 °C [15]. Meteorological data were obtained from the Botucatu weather station between January and May of 2024 and 2025. The variables analyzed included daily global radiation (RgMj_Tot, MJ m^−2^); wind speed (m s^−1^), mean, maximum, and minimum air temperature (°C); mean, maximum, and minimum relative humidity (%); and daily precipitation (mm).

In 2024, monitoring covered 181 days, with mean radiation of 17.7 MJ m^−2^, wind speed of 0.96 m s^−1^, mean temperature of 22.8 °C (30.7 °C maximum, 18.6 °C minimum), mean relative humidity of 68.8%, and total precipitation of 1016 mm. In 2025, 175 days were monitored, with averages of 18.9 MJ m^−2^ radiation, 1.08 m s^−1^ wind speed, mean temperature of 21.5 °C (28.6 °C maximum, 17.2 °C minimum), mean relative humidity of 73.1%, and accumulated rainfall of 1202 mm. These data supported the climatic characterization of the experimental period and the interpretation of results.

### 2.2. Experimental Design and Treatments

The experiment was arranged in a split-plot factorial design, with crop cycle (2024 and 2025) assigned to main plots and photoperiod management (with and without supplemental lighting) to subplots. Ten randomized blocks were established, with two Comet plants per subplot, totaling 40 experimental units. A buffer of 30 plants was maintained between systems, cultivated and managed but excluded from analyses.

### 2.3. Crop Management

‘Comet’ hop plants were spaced 3.2 × 1.0 m and trained on a V-trellis system with sisal strings extending from the soil to the top of a 6.0 m structure. Eucalyptus posts (7.5 m, 1.5 m buried) supported the trellis, connected with steel wires to secure the strings. Drip irrigation was installed with double drip lines, emitters spaced 50 cm apart, and a flow rate of 0.5 L h^−1^, adjusted according to crop water demand at each phenophase [16].

Fertilization was guided by soil analyses conducted prior to each season in the Soil and Environmental Resources Department laboratory (FCA/UNESP). Soil chemical characterization for both treatments is detailed for 2024 and 2025. Recommendations were based on the Fertilizer Guide: Hops (Gingrich, Hart and Christensen, 1994) [17] and Aquino, Teixeira & Macedo (2022) [16]. Nitrogen was supplied as urea (400 kg ha^−1^), phosphorus as Yoorin Master^®^, Yoorin Fertilizantes, Poços de Caldas, Minas Gerais, Brasil, (294.11 kg ha^−1^, applied only in illuminated plots), and potassium as potassium sulfate (550–700 kg ha^−1^). Additionally, 1 kg of compost per plant (produced at FCA/UNESP) was incorporated, with the following chemical composition: N (0.81%), P_2_O_5_ (0.37%), K_2_O (0.09%), Ca (1.25%), Mg (0.17%), organic matter (24%), organic carbon (14%), C/N ratio 17:1, and pH 5.9. Green manures (vetch, black oat, forage radish) were also intercropped and managed as cover biomass.

Phytosanitary management included Fitoneem^®^, DalNeem, Itajaí, Santa Catarina, Brasil, (40 mL per 20 L water) for general insect and mite control, Abamex^®^, Sumitomo Chemical, Arapongas, Paraná, Brasil, (20 mL per 20 L water) for spider mites (*Tetranychus urticae*, *Tetranychus* spp.), and Regent^®^, BASF, Guaratinguetá, São Paulo, Brasil, for leaf-cutter ants (*Atta* spp.).

### 2.4. Photoperiod Management

Supplemental lighting was provided by LED floodlights, Elgin, Mogi das Cruzes, Brasil, (50 W, 10,000 lm, 6500 K, 400–700 nm spectrum) installed at 5.5 m, directed toward the plants. During the vegetative phase, the photoperiod was extended to 17 h daily, with automatic activation 30 min before sunset until the set duration was reached. The mean photosynthetic photon flux density (PPFD) at canopy level was 1.31 µmol m^−2^ s^−1^, following Acosta-Rangel et al. (2024) [18], who demonstrated that low-intensity night-break lighting (0.47 µmol m^−2^ s^−1^) effectively delayed flowering and enhanced vegetative growth of hops in subtropical environments. A total of 32 plants were illuminated in a 96 m^2^ area, resulting in 104.2 lm m^−2^ and 333.3 lm per plant. Lighting was maintained until the end of the vegetative stage (approximately 2 months), then suspended to allow the natural photoperiod (11 h 30–45 min) to trigger flowering. Control plants remained under natural light.

### 2.5. Traits Evaluated

The following traits were assessed: phenological development, productive performance, and hop quality traits (aroma and bitter compounds).

### 2.6. Phenology and Growing Degree Days

Crop cycles were monitored in 2024 and 2025. Phenophases were defined as: budbreak (from shoot emergence to full leaf expansion), mature leaves and leaf senescence, corresponding to the vegetative phase, and inflorescence emergence, full inflorescence (pistil development and bract initiation), cone development (bract closure, immature cones), and mature cones (harvest stage), corresponding to the reproductive phase.

Growing degree days (GDD °C) were also calculated; the calculation was done using the equation:

$$GDD=\left(\frac{Tmax-Tmin}{2}\right)-Tbas$$

where “Tmax” is the maximum temperature of the day, “Tmin” is the minimum temperature, and “Tbas” is the basal temperature, and the value used was 5.6 °C. This value was defined based on research conducted by Barreto and Pilau (2023) [19], which defined basal temperature values for calculating hop degree days in subtropical and tropical regions. Subsequently, the correlation (r) between the cycle duration and the GDD values was performed. A spreadsheet was used to perform the GDD and r calculations.

### 2.7. Morphometric Traits

At harvest, data collected included first cone insertion height (cm), lateral branch length (cm), plant height (cm), fresh cone mass (g), fresh mass per cone (g), dry cone mass (g), and cone length (cm). Cones were dried at 40 °C in forced-air ovens to 10% moisture (~48 h).

### 2.8. Essential Oil Yield and Profile

The essential oil was distilled from 50 g of dried cones by Clevenger hydrodistillation (2 L flask, 1 L deionized water, for 180 min). Yields were expressed as oil mass relative to dry matter (%). The qualitative analysis of essential oils was carried out using a Thermo Scientific gas chromatograph (TRACE 1300 Series GC model) coupled to a mass spectrometer (ISQ 7000 model) and equipped with an autosampler (Triplus RSH), both from Thermo Fisher Scientific, Waltham, MA, USA. Essential oil samples (1 mg each) were diluted in 1 mL of ethyl acetate (chromatographic grade), and 1 µL of this solution was injected. The injector was maintained at 220 °C and operated in split mode with a ratio of 20:1. Compound separation was achieved using a TR-5 MS capillary column (30 m × 0.25 mm × 0.25 µm) with helium (99.999% purity) as the carrier gas at a flow rate of 1.0 mL min^−1^ under the following temperature program: 60 °C to 240 °C at 3 °C min^−1^. The mass spectrometer (MS) operated in full-scan mode with electron ionization (70 eV) and an acquisition range of 40–450 *m*/*z*. The interface temperature was set at 240 °C and the detector at 250 °C. Compound identification was based on comparison of the obtained mass spectra with the National Institute of Standards and Technology (NIST 14) and Flavour & Fragrance Natural & Synthetic Compounds (FFNSC3) libraries, as well as on comparison of linear retention indices (LRI) with literature data [20]. Linear retention indices were determined by injecting a series of *n*-alkanes (C_9_–C_24_, Sigma-Aldrich, 99%, Sigma Aldrich Co., (St. Louis, MO, USA). under the same chromatographic conditions as the samples, applying the Van den Dool and Kratz (1963) equation [21]. Quantitative analysis was performed using GC-FID (GC-2010/AOC-20i, Shimadzu Corporation, Kyoto, Japan). Compound separation was performed on a RA-5 capillary column (30 m × 0.25 mm × 0.25 µm) with helium (99.999% purity) as the carrier gas at a flow rate of 1.0 mL min^−1^, following the same temperature program as the GC-MS system. Area normalization was used to determine the percentage of essential oil components without using correction factors.

### 2.9. Sensory Categories and Aroma Wheel

GC-MS-identified volatiles were assigned to sensory categories using the aroma wheel, with intensities (1–5) estimated from chromatographic peak areas, following Herkenhoff et al. (2024) [22].

### 2.10. Determination of α- and β-Acids

For acid extraction, 2.5 g samples of ground hop (fine powder) were used, to which 50.0 mL of methanol were added. The mixture was shaken for 30 min at room temperature, followed by a 10-min resting period. Subsequently, the solution was filtered through a Millipore membrane (0.45 μm) to remove particulate matter. A 50 μL aliquot of the filtrate was transferred to a 25 mL volumetric flask, and the volume was completed with a methanolic NaOH extracting solution (0.5 mL of 6 M NaOH in 250 mL of methanol). The resulting solution was placed in a quartz cuvette (1 cm optical path) for reading in a UV-Vis spectrophotometer, using 50 μL of methanol in 25 mL of methanolic NaOH as blank [23]. Absorbance readings were performed at wavelengths of 275, 325, and 355 nm, and the α- and β-acid contents (%) were calculated according to the following equations:

$$Total\;\alpha -acids\left(\%\right)=\left(-51.26*A_{\left\{355\right\}}\right)+\left(73.79*A_{\left\{325\right\}}\right)-\left(19.07*A_{\left\{275\right\}}\right)$$

$$Total\;\beta -acids\left(\%\right)=\left(55.27*A_{\left\{355\right\}}\right)-\left(47.59*A_{\left\{325\right\}}\right)+\left(5.10*A_{\left\{275\right\}}\right)$$

### 2.11. Statistical Analysis

Phenological data were tabulated and analyzed descriptively. Morphometric and phytochemical traits (α- and β-acids) were subjected to ANOVA (*p* < 0.05), with means compared by Tukey’s test (*p* < 0.05). Statistical analyses were conducted using AgroEstat^®^, 1.1.0.712 and chemical profiles were tabulated for interpretation.

## 3. Results and Discussion

This study presents the first systematic evaluation comparing contrasting artificial lighting systems in hop cultivation under subtropical conditions in Brazil. Phenological, yield, and phytochemical traits of the Comet cultivar were assessed under conditions with and without supplemental lighting across two consecutive production cycles. This approach enabled precise quantification of agronomic and biochemical responses and advanced the experimental understanding of a cultivation practice already implemented by local growers.

The results presented below encompass climatic conditions, phenological stage duration, yield performance, and cone chemical composition, linking environmental, physiological, and biochemical variables that define the potential of supplemental lighting as a management strategy at lower latitudes. Climatic data from 2024 and 2025 revealed clear associations with hop phenology under contrasting photoperiod regimes (Table 1).

In 2024, mean global radiation was 17.7 MJ m^−2^, with an average temperature of 22.8 °C (maximum 30.7 °C) and cumulative rainfall of 1016 mm. Under these conditions, plants without supplemental lighting completed their phenological cycle in 91 days, while those under an extended photoperiod maintained vegetative growth longer, reaching 108 days. In 2025, radiation was higher (18.9 MJ m^−2^), mean temperature slightly lower (21.5 °C), and precipitation greater (1202 mm), naturally extending the cycle to 97 days without lighting and to 113 days with lighting.

These results demonstrate that, although interannual climatic variability strongly influences the duration of budburst, elongation, flowering, and maturation, supplemental lighting is decisive for maintaining plants in a vegetative state for longer, thereby promoting phenological uniformity and productive potential. The prolonged cycle reflects enhanced vegetative growth and delayed flowering. As highlighted by Acosta-Rangel, Agehara, and Rechcigl (2024) [18], photoperiod extension can even inhibit floral induction in hops, underscoring the critical role of supplemental light in modulating crop phenology.

By extending the phases of shoot elongation, floral initiation, and cone development, supplemental lighting positively affected both yield and technological quality, ensuring greater uniformity of maturation and higher accumulation of secondary metabolites. This effect is particularly relevant at lower latitudes, where natural photoperiods are insufficient to sustain longer cycles, as observed in traditional hop-growing regions.

Table 2 presents the accumulated growing degree day (GDD) values corresponding to the vegetative and reproductive phenological phases, as well as their total sum representing the complete growth cycle for the 2024 and 2025 harvests. The table also includes Pearson correlation coefficients between cycle duration and GDD accumulation for each harvest.

According to Fagherazzi (2020) [24], the accumulation of growing degree days (GDD) is a key determinant of hop development, from the onset of bud emergence to cone maturation. In the 2024 season, both treatments required more than 2000 GDD to complete the growth cycle, totaling 2589.16 °C in light-supplemented plants and 2092.75 °C in non-supplemented plants. In the 2025 season, the cumulative heat requirement was 2115.77 °C for supplemented plants and 1728.29 °C for those grown under natural photoperiod conditions.

Across both harvests, plants exposed to supplemental lighting, which ensured attainment of the critical photoperiod, consistently required greater thermal accumulation to complete their developmental stages. Photoperiod management was decisive in extending the crop cycle, particularly the reproductive phase associated with cone formation and maturation. Consequently, higher energy input was necessary for these phenological transitions. Conversely, plants cultivated without supplemental light accumulated fewer degree days, which shortened the reproductive period and ultimately reduced yield potential.

As reported by Marovt (2007) [25], the total GDD required for hop development varies among cultivars, typically ranging between 1751 °C and 2900 °C. This agrees with the present findings, in which similar values were recorded. Gonsaga (2021) [26], when evaluating multiple cultivars, observed a range of approximately 1500 to 2200 GDD between early and late maturing varieties, emphasizing that reduced thermal accumulation is often associated with earlier phenology and lower cone yield due to limited reproductive development.

The correlation coefficients (r) between thermal accumulation and crop duration were positive (r > 0) in all cases, with stronger associations observed during the second season (values approaching 1), indicating very strong relationships. As described by Miot (2018) [27], positive coefficients denote direct correlations, supporting the conclusion that cycle duration and heat demand are tightly coupled physiological parameters in hop growth.

Analysis of variance confirmed significant effects (*p* < 0.01) of supplemental lighting on several traits. Light significantly increased the number of lateral branches (NLB), lateral branch length (LBL), cone length (CL), and plant height (PH), while the insertion height of the first cone (IHC) remained unaffected. For yield traits, fresh plant mass (FPM), fresh cone mass per plant (FM), dry cone mass per plant (DM), and dry mass per cone (DMC) were all significantly higher (*p* < 0.01) under supplemental lighting. Phytochemically, α-acid and essential oil contents increased significantly (*p* < 0.01), while β-acid levels showed no difference. Year and year × light interactions were mostly non-significant, except for LBL, which responded to both main and interaction effects.

Overall, supplemental lighting had a pronounced impact on hop growth, proving to be a key factor for agronomic performance under subtropical conditions. Traits such as NLB, PH, and CL were significantly enhanced under lighting (Table 3), reflecting greater vegetative vigor and a more balanced architecture that optimizes light interception and biomass accumulation. These responses indicate that supplemental light not only enhances vertical growth but also stimulates lateral branching, which is critical for maximizing yield potential.

The insertion height of the first cone (IHC) is an important agronomic trait in hops, as it reflects plant architecture and indicates the potential for cone distribution and yield. Lower values denote cone formation closer to the soil, increasing the number of fertile nodes and enhancing productivity. In this study, supplemental lighting significantly reduced IHC to 99.0 cm compared with 125.7 cm in plants without lighting. Similarly, Fortuna et al. (2023) [28] reported values ranging from 1.51 to 1.95 m across cultivation systems and varieties in Brazil, with no significant differences, but highlighted that lower IHC values were associated with higher cone yields in cultivars such as ‘Chinook’, ‘Cascade’, and ‘Columbus’, reinforcing the link between this morphological trait and crop performance.

The number of lateral branches (NLB) was strongly influenced by lighting, averaging 70.05 under supplemental lighting versus only 13.0 without. This marked increase reflects the positive effect of photoperiod manipulation on fertile branch emission, expanding the number of reproductive sites and boosting yield potential. According to Leles et al. (2023) [29], enhanced lateral branching is directly associated with greater cone production, especially after plants reach the top of the trellis, when resources are redirected to branch emission.

Plant height (PH) also differed significantly, with plants under lighting reaching 590.94 cm compared with 468.60 cm without. Photoperiod extension promoted greater vegetative growth and delayed floral induction, increasing radiation interception and the number of fertile nodes. Comparable results were reported by Neves et al. (2024) [30], who found significant variation among cultivars grown under subtropical conditions: ‘Comet’ (561.12 cm) was the tallest, followed by ‘Chinook’ (512.04 cm) and ‘Nugget’ (498.50 cm), while ‘Cascade’ (442.12 cm) and ‘Columbus’ (375.87 cm) were shorter. In that study, Comet’s greater height was also linked to improved water-use efficiency, indicating superior biomass conversion and adaptability under subtropical conditions.

Cone length (CL) also responded positively, averaging 3.49 cm with lighting versus 2.84 cm without. Photoperiod extension thus promoted reproductive development. Cernea and Vâtcă (2013) [31] reported a positive correlation between cone length and fresh mass, suggesting that this trait contributes to biomass accumulation. However, they noted that cone dry mass is a more robust and reliable indicator of productivity than length alone.

Lateral branch length (LBL) proved especially sensitive to the interaction between lighting and crop year, with averages of 78.98 cm in 2024 and 97.09 cm in 2025, compared with 42.80 cm and 43.60 cm, respectively, in plants without lighting (Table 4).

These results indicate that the response to photoperiod extension is robust and reinforced by the physiological maturity of hops, a perennial species. The combination of additional light stimulus and reserve accumulation across production cycles enhanced branch elongation, improving light interception and the availability of reproductive sites. Thus, supplemental lighting acts synergistically with plant ontogeny, boosting vegetative vigor and yield potential in perennial systems, as also noted by Leles et al. (2025) [32].

Supplemental lighting produced striking gains in biomass and cone yield, with fresh plant mass (FPM) of 6156.40 g compared with 505.85 g, fresh cone mass (FCM) of 1625.62 g compared with 39.73 g, and cone dry mass (CDM) of 374.61 g compared with 9.75 g in non-supplemented plants (Table 5).

These gains confirm that photoperiod extension enhances both vegetative and reproductive growth, ensuring greater biomass accumulation and more abundant cone formation. Similar results were reported by Leles et al. (2023) [29], who observed significant increases in cone number, fresh mass, and yield in cultivars exposed to 17 h of daily light under subtropical conditions. The authors emphasized that supplemental light, by delaying premature floral induction, strengthens vegetative vigor and promotes lateral branching, thereby expanding the availability of fertile sites. Together, these findings consolidate supplemental lighting as a decisive practice for enabling hop production in subtropical regions, achieving yields comparable to those in traditional growing areas.

Regarding chemical composition, supplemental lighting increased α-acid and essential oil contents by approximately 27.5% and 41.8%, respectively, compared with non-supplemented plants. In contrast, β-acid levels remained similar between treatments, with only a slight increase under lighting (Table 6).

The α-acid content, the main contributor to beer bitterness and stability, was significantly higher in plants under photoperiod management (11.92%) compared with those without supplemental light (9.35%). This increase indicates that day-length extension stimulates the biosynthesis and accumulation of these compounds, reinforcing the potential of ‘Comet’ as a dual-purpose hop, capable of providing high bitterness without compromising its aromatic profile [33].

For β-acids, no statistical differences were detected between treatments, with values of 2.88% under supplemental lighting and 2.34% without. A slight upward trend under light extension may contribute to secondary sensory attributes, such as smoother bitterness and antimicrobial activity in wort. Similar values were reported by Sabino et al. (2025) [14] for ‘Comet’ cultivated under subtropical conditions without supplemental lighting, where α-acids averaged 10.54% and β-acids 4.30%, confirming the genetic potential of this cultivar for expansion into new production regions.

Across both seasons, α-acid levels were consistently higher in plants grown under supplemental lighting. However, even in the absence of this management practice, values remained within the reference range for Comet under temperate conditions (9.0–12.0%). For β-acids, concentrations also approached the expected range of 3.0–6.1% [34]. The essential oil content obtained in this study was consistent with the general range of 0.5% to 3.0% of cone dry mass reported by Almaguer et al. (2014) [35].

GC-MS/FID analysis allowed the identification of 31 compounds in the essential oils (Table 7) across the evaluated harvests, under both lighting conditions (with and without supplemental light). The identified compounds included monoterpene and sesquiterpene hydrocarbons, esters, oxygenated sesquiterpenes, and oxygenated monoterpenes. No significant effects of the evaluated factors or their interaction were observed (*p* < 0.01).

Chemical profiling of the essential oils revealed high stability across years and management regimes, with no significant variation in the overall distribution of compound classes. Monoterpene hydrocarbons predominated (69.74–79.03%), with β-myrcene as the principal constituent. Concentrations ranged from 76.36% to 69.74% in 2024 (without and with supplemental light, respectively) and from 79.03% to 73.25% in 2025, confirming β-myrcene as the primary chemical marker of hops.

According to Durello et al. (2019) [6] and Teixeira (2024) [36], β-myrcene content can vary widely among cultivars (0.5–70%), generally representing the dominant volatile compound and serving as an indicator of hop quality and freshness. However, its high volatility, susceptibility to oxidation, and low water solubility limit its stability and sensory persistence, resulting in a mild and short-lived “hoppy” aroma [37,38].

Sesquiterpene hydrocarbons remained stable (10.09–14.87%), dominated by (*E*)-caryophyllene (5.58%), α-selinene (3.47%), and β-selinene (2.33%). In contrast to monoterpenes, sesquiterpenes exhibit lower volatility and greater oxidative stability [35]. Comparable patterns were observed by Sabino et al. (2025) [14] and Campos et al. (2025) [39] in ‘Comet’ hops cultivated under subtropical conditions, where β-myrcene was the predominant compound, followed by (*E*)-caryophyllene and intermediate levels of α- and β-selinene. These sesquiterpenes have also been recurrent in Brazilian-grown hops, reinforcing their role as potential chemical markers of adaptation to local environments [40].

Esters varied within a narrow range (5.17–7.06%), with geranyl isobutyrate as the main representative (1.91%), while oxygenated monoterpenes and sesquiterpenes remained below 1.3%. Ketones and unidentified compounds exhibited only minor fluctuations, without significant impact on the overall oil profile. From a brewing perspective, such compositional consistency is desirable, as it minimizes the risk of undesirable aromatic deviations during processing.

Overall, the ‘Comet’ cultivar displayed a highly stable aromatic profile across years and light regimes, characterized by β-myrcene dominance complemented by sesquiterpenes and esters. The resilience of its terpenoid metabolism under subtropical conditions ensures reproducible chemical composition and reliable sensory quality across harvests.

**Figure 2:**
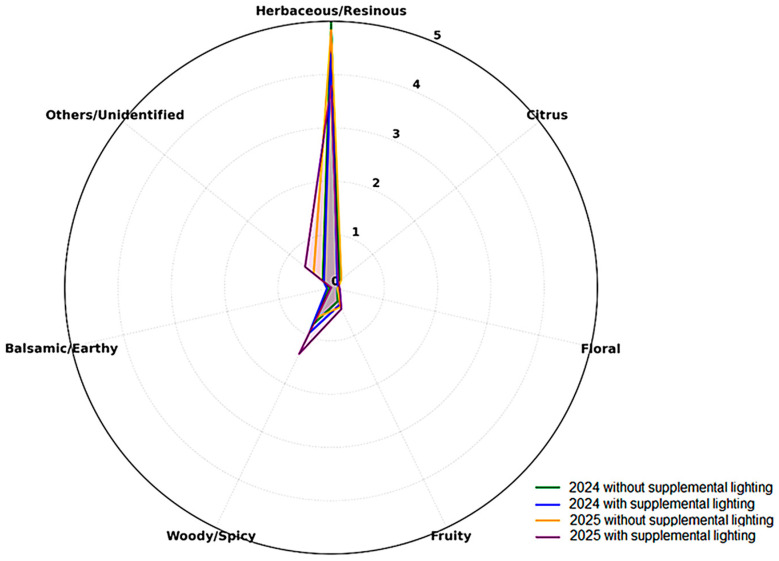
Radar chart showing the relative distribution of the main olfactory notes identified in *Humulus lupulus* L. cones from the 2024 and 2025 harvests cultivated with and without supplemental lighting. Legend: Colors indicate treatments: green—2024 without supplemental lighting; blue—2024 with supplemental lighting; orange—2025 without supplemental lighting; purple—2025 with supplemental lighting.

The aroma wheel (Figure 2), based on Herkenhoff et al. (2024) [22], further illustrates the common aromatic base of all treatments, defined by the herbaceous/resinous dimension driven by high β-myrcene abundance, reinforcing the green–resinous freshness as a hallmark of hops cultivated under subtropical environments.

The volatile profile of *Humulus lupulus* L. cones exhibited consistent patterns of secondary metabolite formation, irrespective of photoperiod management. Across all treatments, β-myrcene was the predominant compound, sustaining the herbaceous, resinous, and fresh character typical of hops cultivated at lower latitudes. This predominance defines a stable sensory identity in which vegetal freshness remains central to the overall aromatic expression.

These results corroborate those of Campos et al. (2025) [39], who demonstrated that in ‘Comet’ hops subjected to different hydrodistillation durations, the herbal descriptor was the most prominent among treatments, reflecting the high monoterpene content, particularly β-myrcene, responsible for fresh and green notes. Similarly, Bonfim et al. (2025) [41] reported stability in the volatile composition of ‘Fuggle’ hops grown under subtropical conditions, identifying β-myrcene, (*E*)-β-farnesene, α-selinene, β-selinene, and (*E*)-caryophyllene as key constituents. β-Myrcene consistently emerged as the dominant metabolite over three consecutive harvests (2021–2023), imparting resinous and herbaceous notes complemented by woody, floral, and citrus nuances, thereby maintaining aromatic typicity despite environmental variability. Such stability reflects metabolic resilience and ensures predictable sensory quality.

Sesquiterpenes, including (*E*)-caryophyllene, and selinene isomers, were recurrent across years and treatments, reinforcing woody, spicy, and peppery dimensions. Although their relative proportions varied, their consistent occurrence alongside β-myrcene established a shared aromatic framework, contributing to both typicity and complexity. Esters imparted subtle fruity notes that softened the dominant green freshness, while oxygenated volatiles such as linalool, methyl geranate, and geranyl acetate introduced floral and citrus nuances, enhancing aromatic balance. This interaction suggests that hops cultivated under subtropical conditions maintain a fundamental equilibrium among herbaceous, floral, fruity, and spicy dimensions [22].

Collectively, these findings demonstrate that all treatments preserved a stable aromatic identity, characterized by herbaceous and resinous freshness enriched with complementary woody, floral, and fruity layers. This convergence indicates that hop cultivation under subtropical conditions, whether with or without supplemental lighting, supports a consistent sensory profile, providing the aromatic stability and technological reliability required by the brewing industry.

## 4. Conclusions

Supplemental lighting proved decisive for hop production under subtropical conditions, driving significant changes in phenological development, yield performance, and phytochemical quality of the Comet cultivar. Artificial photoperiod extension prolonged vegetative growth, enhanced lateral branching, increased plant height, and lengthened cones, traits directly linked to higher yield potential.

The observed gains in biomass and cone productivity, coupled with higher α-acid and essential oil contents, position supplemental lighting as a strategic tool for maximizing yield while ensuring brewing quality. Moreover, the stability of the volatile profile, dominated by β-myrcene and complemented by sesquiterpenes and esters, demonstrates that Comet maintains a consistent aromatic identity regardless of management regime.

Taken together, these results establish supplemental lighting as an effective strategy for adapting hop cultivation to lower latitudes, ensuring phenological regularity, yield improvement, and stable phytochemical quality. This advancement strengthens the foundation of hop production in Brazil, reducing import dependence and expanding opportunities for Comet across both craft and industrial brewing markets.

## Figures and Tables

**Table 1:** Duration of phenological stages (days) of hop (*Humulus lupulus* L.) cv. ‘Comet’ across harvests under different photoperiod management treatments.

Harvest 2024				Harvest 2025		
Treatments	Vegetative	Reproductive	Total	Vegetative	Reproductive	Total
With supplementation	31	77	108	33	80	113
Without supplementation	31	60	91	33	64	97

**Table 2:** Growing degree day (°C) values for the phenological phases, vegetative, reproductive and for the total cycle, and the correlation of GDD (r) with the total duration of the cycles.

Harvest 2024					Harvest 2025			
Treatments	Vegetative	Reproductive	Total	r	Vegetative	Reproductive	Total	r
With supplementation	1237.55	1367.26	2589.16	0.85	616.525	1519.28	2115.77	0.99
Without supplementation	1141.72	972.37	2092.75	0.79	616.525	1131.80	1728.29	0.99

**Table 3:** Mean values of insertion height of the first cone (IHC, cm), number of lateral branches (NLB), plant height (PH, cm), and cone length (CL, cm) in hop plants cultivated with and without supplemental lighting.

Treatments	IHC	NBL	PH	CL
With supplementation	99.00 b	70.05 a	590.94 a	3.49 a
Without supplementation	125.70 a	13.00 b	468.60 b	2.84 b
C.V. (%)	20.80	11.85	5.76	10.48

Means followed by the same letter do not differ significantly according to Tukey’s test (*p* < 0.05). C.V. (%) = Coefficient of variation.

**Table 4:** Mean values of lateral branch length (LBL, cm) in hop plants grown with and without supplemental lighting across two harvests (2024 and 2025).

	LBL	
	With Supplementation	Without Supplementation
Harvest 2024	78.98 Ab	42.80 Ba
Harvest 2025	97.09 Aa	43.60 Ba
C.V. (%)	5.42	

Means followed by uppercase letters (harvests) and lowercase letters (managements) do not differ statistically according to Tukey’s test (*p* < 0.05). C.V. (%) = Coefficient of variation.

**Table 5:** Mean values of fresh plant mass (FPM, g), fresh cone mass (FCM, g), and cone dry mass (CDM, g) in hop plants cultivated with and without supplemental lighting.

Treatments	FPM	FCM	CDM
Whit supplementation	6156.40 a	1625.62 a	374.61 a
Without supplementation	505.85 b	39.73 b	9.75 b
C.V. (%)	4.06	2.77	13.63

Means followed by the same letter do not differ significantly according to Tukey’s test (*p* < 0.05). C.V. (%) = Coefficient of variation.

**Table 6:** Mean values of α-acid content, β-acid content, and essential oil content (%) in hop plants grown with and without supplemental lighting.

Treatments	α-Acid	β-Acid	Essential Oil Content
With supplementation	11.92 a	2.88	1.90 a
Without supplementation	9.35 b	2.34	1.34 b
C.V. (%)	3.21	2.16	4.93

Means followed by the same letter do not differ significantly according to Tukey’s test (*p* < 0.05). C.V. (%) = Coefficient of variation.

**Table 7:** Identification and quantification of compounds and associated aroma notes in hop cones (*Humulus lupulus* L.) from the 2024 and 2025 harvests under two photoperiod management regimes.

					Relative Area (%)					
N°	Substance					HARVEST 2024		HARVEST 2025		Main Fragment ions (*m*/*z*)
			R.T.	LRI Exp	LRI Lit	Without Supplementation	With Supplementation	Without Supplementation	With Supplementation	
1	Isobutyl isobutyrate		5.36	914	908	0.03	0.02	0.15	0.06	116, 43, 56, 73, 88
2	α-Pinene		5.43	936	932	0.17	0.07	0.12	0.09	136, 93, 91
3	Isoamyl propionate		6.39	973	960	0.61	0.32	0.58	0.35	57, 70, 87, 115
4	β-Pinene		6.69	985	974	1.19	0.67	1.00	0.93	136, 69, 93
5	Myrcene		6.92	992	988	70.42	65.81	74.14	69.32	136, 93, 69, 41
6	Isoamyl isobutyrate		7.82	1015	1007	0.79	0.13	0.70	0.38	136, 69, 93
7	Methyl heptanoate		8.06	1027	1021	0.30	0.27	0.25	0.15	143, 43, 74, 87
8	Limonene		8.29	1034	1024	0.35	0.24	0.24	0.25	136, 68, 93
9	β-Phellandrene		8.39	1037	1025	0.67	0.48	0.49	0.50	136, 121, 93,
10	(*E*)-β-Ocimene		8.81	1048	1044	3.56	2.47	3.03	2.16	136, 93, 79, 105, 121
11	2-Nonanone		11.54	1098	1087	0.04	0.01	0.09	0.07	136, 69, 93, 121,
12	Linalool		10.88	1106	1095	0.63	0.79	0.54	0.57	154, 71, 93, 121, 136
13	Methyl octanoate		11.29	1128	1123	0.32	0.38	0.23	0.24	143, 43, 74, 87
14	Methyl nonanoate		15.49	1229	1223	0.31	0.46	0.09	0.15	157, 43, 74, 87
15	2-Undecanone		17.37	1300	1293	0.21	0.26	0.13	1.37	170, 43, 58, 71
16	Methyl geranate		20.14	1328	1322	0.49	0.88	0.55	0.64	182, 69, 41, 114, 123, 151
17	Geranyl acetate		22.50	1384	1379	0.88	1.03	0.86	1.13	154, 69, 93, 136
18	(*E*)-Caryophyllene		24.14	1425	1417	4.42	5.80	5.45	6.66	204, 93, 133, 161
19	α-Humulene		25.63	1462	1452	0.23	0.39	0.15	0.30	204, 93, 80, 121
20	Geranyl propionate		26.22	1475	1476	0.80	0.91	0.47	0.57	154, 69, 57, 93, 136
21	β-Selinene		27.03	1496	1489	1.84	2.41	2.15	2.92	204,105, 93, 79, 161, 189
22	α-Selinene		27.17	1503	1498	2.41	3.53	2.46	3.50	204, 189, 133, 107, 93
23	Geranyl isobutyrate		27.23	1514	1514	0.01	0.03	0.07	0.13	224, 69, 93, 121, 136
24	γ-Cadinene		27.47	1521	1522	1.91	2.66	1.29	2.04	204, 161, 105, 119
25	δ-Cadinene		28.22	1525	1522	0.21	0.36	0.10	0.16	204, 105, 119, 161
26	α-Cadinene		28.81	1539	1537	0.05	0.05	0.06	0.11	204, 119, 161
27	Selina-3,7(11)-diene		29.08	1549	1545	0.56	1.13	0.41	0.59	204, 161,122,107,91,189
28	Germacrene B		29.79	1567	1559	0.36	0.57	0.34	0.50	121, 93, 105,133, 161,189, 204
29	(2*E*)-Tridecenol		31.26	1577	1568	0.25	0.21	0.07	0.12	207, 180, 82, 68
30	α-Eudesmol		33.40	1668	1652	0.33	0.45	0.29	0.80	222, 59, 93, 149, 189
31	Neo-intermedeol		33.71	1672	1658	0.23	0.32	0.17	0.36	207, 67,82,96
Monoterpene hydrocarbons (%)						76.36	69.74	79.03	73.25	
Oxygenated monoterpenes (%)						0.63	0.79	0.54	0.57	
Sesquiterpene hydrocarbons (%)						10.09	14.27	11.18	14.87	
Oxygenated sesquiterpenes (%)						0.81	0.98	0.53	1.28	
		Esters (%)				6.44	7.06	5.17	5.71	
		Ketones (%)				0.25	0.27	0.22	1.44	
		Unidentified (%				4.42	2.89	3.33	2.88	

R.T.: Retention time; LRIexp: Experimental Linear Retention Index; LRIlit.: Literature Linear Retention Index (Adams, 2017) [20].

## Data Availability

Data are contained within the article.
